# Kidney Diseases and Pregnancy: A Multidisciplinary Approach for Improving Care by Involving Nephrology, Obstetrics, Neonatology, Urology, Diabetology, Bioethics, and Internal Medicine

**DOI:** 10.3390/jcm7060135

**Published:** 2018-06-04

**Authors:** Giorgina Barbara Piccoli, Rossella Attini, Gianfranca Cabiddu

**Affiliations:** 1 Department of Clinical and Biological Sciences, University of Torino, 10100 Torino, Italy; 2 Nephrologie CH Le Mans, 72000 Le Mans, France; 3 Obstetrics, Department of Surgery, University of Torino, 10100 Torino, Italy; rossella.attini@gmail.com; 4 Nephrology, Azienda Ospedaliera Brotzu, 09100 Cagliari, Italy; gianfranca.cabiddu@tin.it

**Keywords:** Kidney diseases, pregnancy, multidisciplinary approach, nephrology, obstetrics, neonatology, urology, diabetology, bioethics

## Abstract

This multidisciplinary series is aimed at offering readers many opportunities to appreciate how a clinical and ethical approach to pregnancy has changed in patients with kidney diseases and with related conditions, including diabetes, hypertension, and immunologic diseases. Furthermore, this series aims to focus on the fact that many issues remain unreslved, that there are enormous gaps in knowledge, and that the bioethical approach needs to integrated in the clinical practice, which would allow for a deeper appreciation of different cultural and religious backgrounds. Much still needs to be done to allow women suffering from all stages of chronic kidney disease (CKD) and those with predisposed conditions, so that they may experience safe pregnancies, starting from an increased awareness of the importance of CKD, even in its early stages, to the detection of risk factors. Women who have experienced preeclampsia or acute kidney injury in pregnancy need to have follow-up checks. The role of urinary infections, kidney stones, and urinary malformations is not fully acknowledged, nor have univocal control schedules and treatment schemas yet been defined for the different kidney diseases. In this regard, the fight for equitable treatment for all women with acute or chronic kidney disease in pregnancy and for the widespread prevention of adverse pregnancy-related and long-term outcomes is ultimately a battle for equitable healthcare.

Pregnancy in dialysis patients is the tip of an iceberg. When, in 1970, professor Confortini reported on the first full-term pregnancy in a patient on haemodialysis, few, if any, nephrologists expected that, some 50 years later, hundreds of women would have conceived and given birth while on haemodialysis [1,2].

It is worth reading Confortini’s paper, which is available online, with its commentary and its handmade graphs that may make us smile today, but at the same time, show us the enormous advances that have been made in medicine, communications, and overall research tools (Figure 1). The World Wide Web did not exist and its revolutionary use in medicine was still to come, but innovation did not wait for the Internet.

Today, the chance that a woman on dialysis will have a baby remains about 1:100 lower than a woman in the general population. In addition, the babies are often born pre-term and are small for their gestational age [2,3,4].

Great advances have been made, but can more be done?

The importance of the lessons that have been learned from these ‘extreme’ cases are not limited to obstetric nephrology, but affect the overall management of dialysis. Advances in dialysis have led to advances in obstetric nephrology, and conversely, advances in obstetric nephrology have improved our understanding of the physiology (or non-physiology) of dialysis and of the level of depuration that can be achieved with renal replacement therapy [5].

These exchanges are very important in nephrology. The field of what is now often called ‘obstetric nephrology’ is almost, by definition, a multidisciplinary one, involving not only nephrologists, obstetricians, and neonatologists, but also many other specialties and competences.

Professor Confortini was not a nephrologist. He belonged to the generation of surgeons who, in the early years of dialysis, in Italy, were often responsible for the care of dialysis patients. He was also one of the first people in this country to perform arterio-venous Cimino-Brescia fistula for hemodialysis.

Reading his paper encourages us to reflect on the importance of being open-minded beyond the ‘usual’ competence of our chosen specialty. His paper still conveys not only his enthusiasm “We think it is worthwhile to report what may be the first case of a female patient undergoing regular dialysis treatment, conceiving, and bringing to full term a perfectly normal pregnancy, and giving birth to a viable and healthy child”, but also his respect for the mother’s wishes. This was a concept that was not widely accepted in the overall paternalistic approach to medicine in early the Seventies, especially in Southern European countries [1].

The paper goes on, “In January 1970 the patient conceived. She did not allow termination of pregnancy, and, because of her excellent clinical condition, perfect mental balance, and her expressed wishes, we decided to try bringing the pregnancy to its full term” [1]. The innovative character of this statement will be better appreciated if we compare it with an article on pregnancy with kidney diseases, which appeared in *The Lancet* at about the same time, and began by stating that, “Children of women with renal disease used to be born dangerously or not at all—not at all if their doctors had their way” [6].

We hope this multidisciplinary series will offer readers many opportunities to appreciate how ‘doctors’ ways’ have changed, while not losing sight of the fact that many issues remain unsolved, that there are enormous gaps in knowledge in all clinical fields, and that the bioethical approach needs to integrated in the clinical practice, allowing for a deeper appreciation of different cultural and religious backgrounds.

Much has to be done to allow women with all stages of chronic kidney disease (CKD) to experience safe pregnancies, starting from an increased awareness of the importance of CKD even in its early stages, to the detection of risk factors, and to the follow-up checks for women who have experienced preeclampsia or acute kidney injury in pregnancy. The role of urinary infections, kidney stones, and urinary malformations is not fully acknowledged, nor have univocal control schedules and treatment schemas yet been defined for the different kidney diseases.

If the lists of what we would like to know are long (Table 1, Table 2 and Table 3), the lists of what we would like to make available might be even longer, and they should be adapted to different settings of care, as it is too early to talk about intensive daily dialysis in a setting where no treatment for acute kidney failure is yet available.

In this regard, it should be kept in mind that the fight for equitable treatment for all women with acute or chronic kidney disease in pregnancy, and for the widespread prevention of adverse pregnancy-related and long-term outcomes, is ultimately a battle for overall equitable healthcare.

This editorial is dedicated to all of the women with a chronic disease who have fought and are fighting to live a full life, and to those who are attempting overcome unexpected barriers, as well as to those who have given up or have been overwhelmed by the difficulties that they have encountered.

Bringing hope should be a part of all treatments.

We know that avoiding unnecessary pregnancy-related deaths is possible, just as we know that this will require a multidisciplinary commitment [7].

Giving birth when suffering from disease is often seen as a life sentence, understanding physiology through pathology, and improving knowledge on kidney diseases are all fascinating challenges. If this series will to add just a little bit to any one of them, our goal will be met.

## Figures and Tables

**Figure 1:**
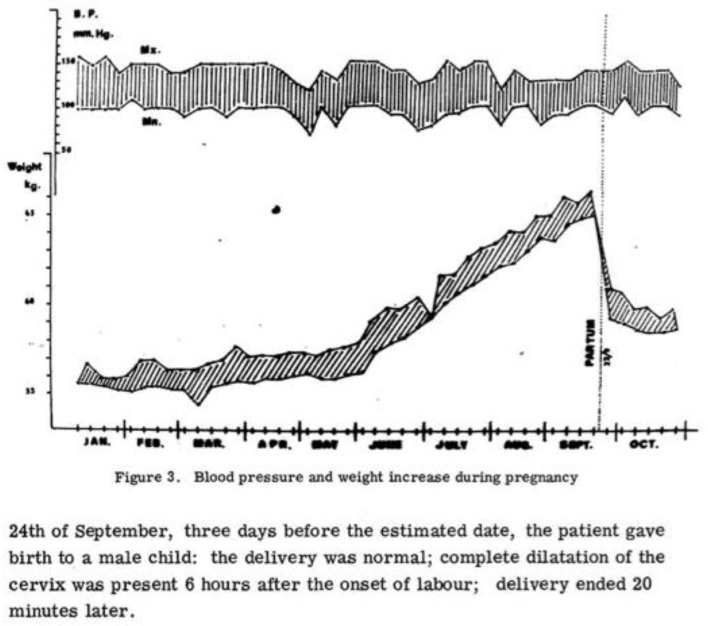
Weight gain during pregnancy on dialysis, from Confortini et al. [1] (Reproduced with permission from ERA-EDTA).

**Table 1:** Some bioethical questions waiting for answers regarding pregnant women with chronic kidney disease (CKD) and related diseases.

Question	Key Points
Is it there a risk threshold for discouraging/forbidding pregnancy in CKD?	None established, context-sensitive, and insufficient evidence of counselling for women with severe or rare conditions.
What is the role of the physician in counselling?	No model of physician–patient interaction is deemed superior to others and the role is context-sensitive (paternalism, therapeutic alliance, informative, etc.).
In pregnancies that are at a high risk for early pre-term delivery, what is the weight of the associated risks for the baby?	This is an example of maternal–foetal conflict: pregnancy in dialysis, with stages 4–5 CKD (proteinuria and hypertension, or with a failing kidney graft) is associated with early pre-term delivery and small babies. The mother’s right to self-determination may conflict with the risk of disability in the offspring.
What is the importance of the risk of impairing residual kidney function (or causing loss of a kidney graft) in a high-risk CKD pregnancy?	This is an issue that has an individual valence (risk of end-stage kidney disease) and a social one (costs of renal replacement therapy and competition for kidney transplantation).
What is the role of genetic counselling and genetic selection in patients with late-onset diseases?	This is the case, for example, for polycystic kidney disease, whose high genetic frequency does not correspond to clinical disease. It generally develops in the 3rd and 4th decade of life, making it difficult to foresee what CKD treatment will be available when the disease becomes clinically overt in the offspring.

**Table 2:** Some clinical questions waiting for answers regarding pregnant women with CKD. BP—blood pressure.

Question	Key Points
What is the interaction between the different renal determinants of pregnancy outcomes (hypertension, proteinuria, and kidney function)?	Each of these factors has been independently associated with adverse pregnancy-related outcomes; their hierarchy, if any, is not known.
Are there specific differences between the various kidney diseases?	Because of the high heterogeneity of CKD, little is known about the effect different kidney diseases have on pregnancy.
What is the role of initial kidney tissue damage in hypertensive or diabetic pregnancies?	Risk factors for preeclampsia are almost all the same as risk factors for the development of CKD. Initial kidney damage may be the final pathway to adverse pregnancy outcomes.
What is the best BP target in CKD or diabetic pregnancies?	The target blood pressure probably depends on control policy. If this is true, stricter controls should allow for safer normalisation.
What is the best frequency of controls in the different CKD stages?	Even if CKD is presently acknowledged as a risk factor in pregnancy, the best policies for follow-up and controls have not yet been established.
When and how should dialysis be started in pregnancy?	The recent literature shows improved results in dialysis patients. When to start dialysis in pregnancy has not been established. An early start may be an option, but it is in contrast with the data suggesting that a later start is safer in all other cases.
What is the role of nutritional management of CKD pregnancies?	Nutritional management can probably compensate for deficits and balance the metabolic derangements of CKD, but its role has to be established.
What should be done in the case of ‘forbidden’ potentially teratogen medications in pregnancy?	There is a wide variety of risks and phenotypes, and the echographic findings are usually available too late.
How should the indications for managing CKD pregnancies be adapted to low- to medium-income countries?	Most of the indications for the care of pregnancies in CKD and related diseases have been defined in high-income countries. Their adaptation to low- to medium-income countries are difficult if not impossible.

**Table 3:** Some clinical questions waiting for answers regarding pregnant women with acute kidney involvement or other diseases potentially involving the kidney.

Question	Key Points
Should all pregnant patients have a kidney-function assessment?	Serum creatinine is an inexpensive test that could allow us to detect, at least, severe kidney diseases.
What is the role of urinary culture control in preventing severe urinary tract infections?	Urinary infections are usually asymptomatic in pregnancy. Their systematic detection is included in some, but not all, of the guidelines for pregnancy care.
What is the best policy of controls to prevent recurrent pyelonephritis or urinary tract infections in pregnancy?	Once detected, urinary tract infections in pregnancy should be treated. The frequency of subsequent controls, the role of echography, and of long-term prophylaxis need to be established.
What is the clinical role of the angiogenic-antiangiogenic biomarkers in pregnancy?	These controversial markers may support the diagnosis of diseases other than preeclampsia, which is usually self- evident.
When a woman has experienced acute kidney injury or preeclampsia, what follow-up should she have in the subsequent pregnancies?	After preeclampsia, the risk for developing a further hypertensive disorder of pregnancy is increased. However, these pregnancies are not uniformly identified as at risk and followed accordingly.
What should the follow-up be for women who have experienced acute kidney injury or preeclampsia after pregnancy?	Even if preeclampsia in pregnancy indicates a long-term risk for CKD, follow-up indications have not been established.
What should be done in the case of ‘forbidden’ potentially teratogen medications in pregnancy?	There is a wide variety of risks and phenotypes. The echographic findings are usually available too late.
How should the indications of the management of preeclampsia, diabetes, and other diseases potentially affecting the kidney in pregnancy be adapted to low- to medium-income countries?	Most of the indications for follow-up and the care of pregnancies in CKD and related diseases have been defined in high-income countries. Their adaptation to low- to medium-income countries will be difficult if not impossible.
